# Biophysical Characterization and Cytocompatibility of Cellulose Cryogels Reinforced with Chitin Nanowhiskers

**DOI:** 10.3390/polym14132694

**Published:** 2022-06-30

**Authors:** Irina V. Tyshkunova, Iosif V. Gofman, Dmitry G. Chukhchin, Alexey V. Malkov, Alexander I. Mishanin, Alexey S. Golovkin, Ekaterina N. Pavlova, Daria N. Poshina, Yury A. Skorik

**Affiliations:** 1 Institute of Macromolecular Compounds of the Russian Academy of Sciences, Bolshoy V.O. 31, 199004 St. Petersburg, Russia; tisha19901991@yandex.ru (I.V.T.); gofman@imc.macro.ru (I.V.G.); rn-ka@mail.ru (E.N.P.); poschin@yandex.ru (D.N.P.); 2 Department of Biology, Ecology and Biotechnology, Northern (Arctic) Federal University Named after M.V. Lomonosov, Severnaya Dvina Emb. 17, 163002 Arkhangelsk, Russia; dimatsch@mail.ru (D.G.C.); a.malkov@narfu.ru (A.V.M.); 3 N. Laverov Federal Center for Integrated Arctic Research of the Ural Branch of the Russian Academy of Science, Severnaya Dvina Emb. 23, 163000 Arkhangelsk, Russia; 4 Almazov National Medical Research Centre, Akkuratova 2, 197341 St. Petersburg, Russia; mishaninssma@yandex.ru (A.I.M.); golovkin_a@mail.ru (A.S.G.); 5 Institute of Chemistry, St. Petersburg State University, Universitetskii 26, Peterhof, 198504 St. Petersburg, Russia

**Keywords:** cellulose cryogels, composite cryogels, chitin nanowhiskers, complex hierarchical morphology, biocompatibility, tissue engineering

## Abstract

Polysaccharide-based cryogels are promising materials for producing scaffolds in tissue engineering. In this work, we obtained ultralight (0.046–0.162 g/cm^3^) and highly porous (88.2–96.7%) cryogels with a complex hierarchical morphology by dissolving cellulose in phosphoric acid, with subsequent regeneration and freeze-drying. The effect of the cellulose dissolution temperature on phosphoric acid and the effect of the freezing time of cellulose hydrogels on the structure and properties of the obtained cryogels were studied. It has been shown that prolonged freezing leads to the formation of denser and stronger cryogels with a network structure. The incorporation of chitin nanowhiskers led to a threefold increase in the strength of the cellulose cryogels. The X-ray diffraction method showed that the regenerated cellulose was mostly amorphous, with a crystallinity of 26.8–28.4% in the structure of cellulose II. Cellulose cryogels with chitin nanowhiskers demonstrated better biocompatibility with mesenchymal stem cells compared to the normal cellulose cryogels.

## 1. Introduction

Cryogelation is a fairly new, developing direction in tissue engineering [1]. The highly porous cryogel structure facilitates cell penetration, contact, and migration, while maintaining chemical and mechanical stability. The process of producing cryogels is very simple and economically viable [2]. In the case of water-insoluble cellulose or chitin cryogels, the inner structure is formed during the regeneration when the macromolecules come closer together and form multiple H bonds, and during freezing and freeze-drying when the ice crystals are formed, determining the pore form and size [3,4]. A set of unique properties—including high water retention, porosity, high pore connectivity, and consistency—make cryogels very similar to natural soft tissues [5,6]. The mechanical stability of cryogels enables their use in in vivo processes [7,8,9].

A large number of different factors affect the structure of cryogels [10]. The freezing temperature affects the cryogel’s structure. Smaller pores can be formed by lowering the freezing temperature [11,12,13]. The freezing rate also affects pore formation [14]. It has been shown that increasing the freezing rate leads to the formation of smaller [15] or even irregular pores [16], depending on how sharply the freezing rate increases. Optimal freezing conditions can be determined by the initial crystallization temperature of the solvent and the freezing point for a particular polymer solution [17].

The concentration and molecular weight of the polymer affect the structure of the resulting cryogel [18,19]. It has been shown that solutions of polymers with a lower molecular weight at the same mass concentration in a hydrogel lead to the formation of larger pores in comparison with hydrogels of polymers with a higher molecular weight [20,21,22]. This is due to an increase in the number of hydrogen bonds and a decrease in the availability of free water. Along with this, the temperature and dissolution time affect the polysaccharide destruction and resulting molecular weight and, therefore, the properties of the cryogels [17].

Biocompatibility is a necessary characteristic for scaffolds. Natural polymers are biocompatible, with minimal immune response. They also stimulate cell-to-cell interaction and have low toxicity, while the use of synthetic polymers can be problematic in terms of injection and infection, and harmful byproducts can be generated during their decomposition [1]. Cellulose-based materials have been proposed for different biomedical applications, such as orthopedic and dental implants, drug carriers, vascular grafts, and wound dressings [23,24,25]. Nordli et al. [26] produced ultrapure CNF-based aerogels using sodium hydroxide followed by TEMPO-mediated oxidation [27]. The evaluation of cytotoxicity on human epidermal keratinocytes and fibroblasts has shown that nanocellulose-based aerogels are as safe as commercial wound dressings.

Hierarchically porous cellulose materials from microcrystalline cellulose (MCC) have been designed for use as bio-based matrices or supporting materials for the synthesis of biocomposites [28]. It was shown that due to the versatility in the hierarchy of pores, the structural and mechanical properties of cellulose scaffolds vary from lightweight, soft materials to hard, stiff, and dense materials. MCC/pectin-based cryogels have been shown to have promising potential for hemostasis [29]. For cellulose cryogels with a pectin content of 40%, the proliferation rates were the highest, and there were no pathological lesions in the organs of mice, suggesting favorable biocompatibility. The results showed less bleeding in the hydrogel-treated liver wound within 3 min. This composite porous material has the potential to be a rapid hemostatic biomedical material.

Cryogels from polyvinyl alcohol (PVA) with different contents of microcrystalline cellulose (10, 30, and 50%) were obtained (microcrystalline cellulose was dispersed in water/NaOH 11/1 *w/w* mixture) in [30]. The resulting cryogels were evaluated as carriers for the biologically active compound vanillin. The study showed an increased percentage of vanillin released with increasing cellulose content in cryogels. Cellulose cryogels prepared by dissolution of MCC in 8 wt% NaOH–water; coagulation in water and lyophilization were used for drug release (procaine hydrochloride) [31]. It was shown that the kinetics and the amount of drug released can be tuned by cellulose concentration. PVA–sodium carboxymethyl cellulose (CMC) gels containing propolis were obtained for application as wound dressings [32]. In tests for the delivery of phenols and flavonoids, which promote antimicrobial activity, a higher content of propolis resulted in faster release, and was sufficient to inhibit *S. aureus* [32].

Injectable cryogels have been developed from CMC and alginate for the minimally invasive delivery of differentiated neuronal networks [33]. These cryogels contain natural laminin, which promotes neuronal adhesion and neurite growth, while having both a high Young’s modulus (protecting the architecture of neurons during injection) and a low macroscopic Young’s modulus (allowing scaffolds to be integrated into soft tissues such as the brain). Using such a scaffold can be a minimally invasive method for repairing damage to the brain tissue [33].

Cryogels based on CMC, PVA, and polyethylene oxide were obtained. To obtain membranes containing *Aloe vera* extract or curcumin, a solution of the corresponding component was mixed with the above polymers before freeze-drying. The gels were able to release curcumin and aloe vera solution, and were bactericidal against *S. aureus* and *E. coli* species [34].

A composite cryogel of nanocellulose/bioactive glass (20/80 ratio) was obtained as a scaffold for bone regeneration, and its properties were compared with those of a cellulose cryogel without added glass [35]. In vitro biocompatibility studies have shown that both cryogels are cytocompatible; none of the cryogels significantly affect metabolic activity or cell growth. Moreover, it has been shown that the resulting materials are suitable for bone regeneration in vivo [35].

Multilayer collagen (Col)–CMC–tricalcium phosphate (TCP) cryogels were obtained in [36]. According to the biodegradation results, Col/CMC and Col/CMC/TCP cryogels can degrade almost 70% of their original form in 4 weeks. The cryogels’ pore diameters (204 μm for Col/CMC, 195 μm for Col/CMC/TCP) and geometry (an anisotropic pore structure with the multilamellar composition of macro- and micropores’ integrity) are optimal for cell proliferation and growth. The evaluation of hemocompatibility showed that the cryogels are non-toxic and compatible with blood, and can potentially be used as materials for the regeneration of hard tissues.

Aerogel microspheres prepared from plant-derived native cellulose nanofibrils by freeze-drying methods can be used as cell culture scaffolds [37]. The results of 3T3 NIH cell culture experiments demonstrated that cellulose aerogel microspheres with porous structures were able to support cell growth and differentiation.

Thus, there is a lot of research in the field of producing cellulose-based cryogel scaffolds, but these are mainly materials obtained from cellulose derivatives or cellulose nanoparticles/nanofibrils. There are certain difficulties in dissolving and producing cellulose-based products due to their crystal structure and strong hydrogen bonds. Previously, we have shown that phosphoric acid can be successfully used as a solvent for cellulose to produce cellulose cryogels [19]. There are a number of variables that determine the structure and properties of cryogels. Changes in cryogel production parameters affect the properties of cryogels for biomedical applications [38]. In this work, we continue our research on the preparation of cellulose cryogels using phosphoric acid as a solvent. The aim of this work is to assess the biocompatibility of cellulose cryogels with the best characteristics, depending on the conditions of their preparation, including the dissolution temperature of cellulose in phosphoric acid, the concentration of cellulose, and the dissolution and freezing times.

## 2. Materials and Methods

### 2.1. Materials

MCC powder with a particle size of 20 microns was purchased from Sigma-Aldrich. The MCC had a weight-average molecular weight (M_w_) of 1 × 10^5^, as determined by gel permeation chromatography (GPC). A cellulose crystallinity of 55.0% was measured by XRD [39] using the program described in [40]. The crab α-chitin (BioLog Heppe GmbH, Landsberg, Germany) used had M_w_ of 5.86 × 10^5^ and dispersity (Ð) of 4.3 [41]. A degree of acetylation of 0.98 was calculated using ^13^C CP-MAS NMR spectroscopy [42]. Concentrated phosphoric acid (85%) was used as the solvent. N,N-dimethylacetamide (DMAc) with a purity of 99.95% was supplied by Vecton (St. Petersburg, Russia). Lithium chloride with a purity ≥ 99% was supplied by Sigma-Aldrich (St. Louis, MO, USA). Pullulan polysaccharide standards with molecular weight ranging from 345 to 805,000 (PSS, Mainz, Germany) were used for GPC.

### 2.2. Methods

For the preparation of the cellulose cryogels, 5% and 10% cellulose solutions in 85% H_3_PO_4_ were prepared. Cellulose powder was mixed with H_3_PO_4_ under various conditions (i.e., different concentrations of cellulose, dissolution times, and temperatures). The procedure for producing cellulose cryogels based on the obtained cellulose solutions was similar to that presented previously [19]. To produce a composite cryogel, the dispersion of chitin nanowhiskers (CNWs) was introduced at the stage of cellulose regeneration from a phosphoric acid solution.

CNWs were obtained via the treatment of crab α-chitin with 3 M HCl at 100 °C for 90 min with constant stirring [43,44]. The CNWs were characterized by a hydrodynamic radius and ζ-potential using a Photocor Compact-Z instrument (Photocor, Moscow, Russia). The source laser wavelength was 659 nm, the temperature was 20 °C, and the detector angle was 90°. Freeze-dried CNWs were redispersed in distilled water using ultrasonic treatment for 20 min, and introduced into the cryogel in an amount of 1–10% relative to cellulose.

The cryogel yield, and the cryogel parameters—such as volume shrinkage, volume variation, specific mass, porosity, and swelling—were measured and calculated in a similar way as in a previous study [19].

The molecular weight of cellulose and cellulose cryogels was determined using the Prominence LC-20 system (Shimadzu, Kyoto, Japan). Chromatographic separation was carried out using two GRAM columns (PSS, Mainz, Germany) (1000 Å, 300 mm × 8 mm) and a GRAM precolumn (50 mm × 8 mm). The column oven and refractive index detector were maintained at 70 °C and 55 °C, respectively. The mobile phase (0.5% LiCl in DMAc) flow rate was 1.0 mL/min, the pressure was 1.9 MPa, and the sample volume was 20 µL. Samples were prepared according to the previously presented method for cellulose [45].

The structure and morphology of cellulose cryogels, including the surface morphology and porosity, along with the water stability of the cryogels (swelling ratio (SR)), were studied according to the methods presented in [19].

To determine the crystallinity of initial MCC and cellulose cryogels, the method of X-ray diffraction (XRD) was used. Diffraction patterns were recorded with an XRD-7000S diffractometer (Shimadzu, Japan) equipped with an adaptor for sample rotation and a polycapillary optical system. The goniometer’s settings were as follows: a θ–θ optical scheme, a scintillation detector, and a monochromator adjusted to a wavelength of 1.5406 Å (Cu Kα1 line). The nonreflecting holder had a rotational rate of 30 rpm. The diffraction Cu-target tube was operated at an accelerating voltage of 50 kV and a current of 30 mA. The intensity was registered in a dispersion 2θ range of 10–70° with a scan rate of 0.5° min^−1^ and a step of 0.02°. Samples were pressed into tablets on a “PP 25” press (Retsch Technology GmbH, Haan, Germany) using a mold with a diameter of 25 mm, with a force of 10 tf. The tablet’s thickness was 1 mm. The degree of crystallinity was determined from diffractograms according to a previously published method [39], using a program specially designed for calculating crystallinity [40].

The mechanical properties of the cryogels were studied on an AG-100kN X Plus universal mechanical test system (Shimadzu Co., Kyoto, Japan). The samples were prepared in the form of tablets with a height and diameter of around 1 and 2 cm, respectively. The compression was performed at room temperature with a compression rate of 5 mm/min. All of the measurements were performed in triplicate.

The biocompatible properties of the cryogel samples were evaluated in in vitro tests using human mesenchymal stem cells (MSCs). Cells were obtained from the adipose tissue of healthy donors. The cells were cultured in an alpha-MEM culture medium (Thermo Fisher Scientific, Waltham, MA, USA) supplemented with 10% fetal bovine serum, 1% L-glutamine, and 1% penicillin/streptomycin solution (Thermo Fisher Scientific, Waltham, MA, USA) in a CO_2_ incubator at 37 °C and 5% CO_2_.

Cylindrical cryogels were cut into fragments that were 10–12 mm in height, and shaped into a rectangular parallelepiped form with a surgical blade. The prepared samples were soaked for 30 min in phosphate-buffered saline (PBS) with the addition of a 2% solution of penicillin/streptomycin, followed by three washings in PBS. Coverslips were treated in 70% ethanol for 10 min, followed by washing three times in PBS. Next, the samples and coverslips were placed in the wells of a 24-well plate. A total of 1 mL of MSC suspension at a concentration of 50,000 cells/mL was added to the wells and co-cultured for 72 h in a CO_2_ incubator. The experiments on the samples and coverslips were carried out in triplicate.

After 3 days, the samples and slides were transferred to the wells of a new plate, washed from culture medium using PBS, and fixed in a 4% paraformaldehyde (PFA) solution for 10 min. After fixation, the samples and slides were washed from PFA in PBS and stained according to a previously developed protocol [46], according to which the samples and slides with cells were first treated with a 0.05% Triton X-100 solution for 3 min, followed by washing three times in PBS. Next, a 1% solution of rhodamine-labeled phalloidin (Thermo Fisher Scientific, Waltham, MA, USA) at a 1:500 dilution in PBS was added to the wells, incubated for 20 min at room temperature in the dark, and then washed five times using PBS. At the final stage, cell nuclei were stained with DAPI (4,6-diamidino-2-phenylindole) dye at a dilution of 1:40,000 and incubated for 40 s, followed by thorough washing with PBS. After staining, the material samples were stored in PBS in the dark at +4 °C. Coverslips with cells from the control wells were fixed on glass slides using a mounting medium and stored in the dark at room temperature.

Stained MSCs on samples and glasses were studied using fluorescent microscopy with a quantitative and qualitative analysis of adherent cells. The cells were visualized using an Axiovert inverted fluorescent microscope (Zeiss Microscopy, Jena, Germany) equipped with a Canon camera. Ten different fields of view were photographed at ×10 and ×40 magnification for each sample. A quantitative analysis was carried out by analyzing photographs at ×10 magnification, and the qualitative analysis was carried out from photographs at magnifications of ×10 and ×40. Statistical data processing was carried out using GraphPad Prism 8 software (San Diego, CA, USA) with the nonparametric Mann–Whitney U test. The results were presented as the mean and standard error (SE).

## 3. Results and Discussion

### 3.1. Preparation and Characterization of Cellulose Cryogels

Cryogels were obtained by dissolving the characterized microcrystalline cellulose in phosphoric acid. According to a previous study [19], the cellulose of similar molecular weight produced the most stable and strong cryogels at a dissolution temperature of 20 ± 2 °C, a cellulose concentration > 3% in the solution, and a dissolution time of 24–48 h. In this study, cryogels were obtained from solutions with a cellulose concentration of 5 and 10% in phosphoric acid, a dissolution time of 24–48 h, and a temperature of 20–40 °C. By varying the conditions for the dissolution of cellulose (i.e., temperature and time of dissolution), cryogels with different characteristics were obtained (Table 1).

It was previously shown that the dissolution of cellulose upon heating to 55 ± 5 °C does not allow for obtaining cryogels, due to the destruction of cellulose in solution at elevated temperatures [19]. In this work, we investigated heating up to 29 ± 1 °C and 39 ± 1 °C. The results showed that the heating of cellulose at a 5% concentration during dissolution in phosphoric acid led to the formation of a powder (samples 3, 5, and 6). An increase in the concentration of cellulose up to 10% under heating (29 °C) made it possible to obtain a cryogel that retained its cylindrical shape (sample 4). At the same time, an increase in temperature up to 29 °C allowed for obtaining a 10% cellulose solution in 24 h, while it previously took 48 h to obtain an 8.4% cellulose solution at room temperature (20 °C) [19]. At 20 °C, cryogels that retained their shape were obtained (samples 1 and 2) in 24 and 46 h, respectively. At the same time, with an increase in the dissolution time, the cellulose yield decreased. This indicates a greater destruction of cellulose during prolonged treatment with phosphoric acid. With the same cellulose concentration, an increase in the dissolution time had a greater effect on the decrease in the yield than an increased temperature (samples 2 and 3). Further increasing the temperature (39 °C) also led to a decrease in the yield of cryogel (samples 5 and 6).

A negative value of volume variation was determined for cryogels that did not retain their shape. A positive result corresponds to the swelling of cellulose after regeneration. The variation in smallest positive volume is attributed to the sample with the highest volume shrinkage (sample 4). The swelling of cellulose hydrogels decreased by three to four times with an increase in the dissolution temperature (29 °C and higher).

The M_w_ of cellulose cryogels decreased by 1.9–7.6 times compared to the initial MCC. Heating led to increased destruction and the greatest decrease in the molecular weight of the cellulose (samples 3–6). Increasing the cellulose concentration to 10% (sample 4) slightly slowed down the dissolution process. Under similar dissolution conditions, the molecular weight of sample 4 (cellulose concentration of 10%) was slightly higher than that of sample 3 (cellulose concentration of 5%). Thus, an increase in the cellulose concentration in the solution decreased the degree of destruction. Samples obtained by dissolving cellulose at 20 °C had the highest M_w_ (samples 1 and 2). An increase in the dissolution time of cellulose (sample 2) led to a greater decrease in M_w_.

The spatial organization of macromolecules during regeneration and drying led to some volume shrinkage of the cryogel samples in comparison to the volume of the initial solution, and a decrease in M_w_ caused by heating and the prolonged exposure to phosphoric acid during dissolution led to greater shrinkage. Cryogels that retained their shape were further characterized (Table 2).

The volume shrinkage of cryogel sample 4 obtained upon heating was the highest (37.3%), and it was the smallest for samples 1 and 2 obtained at 20 °C, at 10.7% and 6.9%, respectively (Table 2). The low volume shrinkage of cryogel samples 1 and 2 resulted in extremely low densities (Table 2), which were almost equal to the theoretical density (0.05 g/cm^3^) calculated for zero volume shrinkage. Due to the greater shrinkage of sample 4, its density was higher (0.144 g/cm^3^) than the corresponding theoretical density (0.10 g/cm^3^). The density increased with increasing cellulose concentration, while the porosity decreased. For the 5% cellulose concentration, a cryogel density of about 0.050 g/cm^3^ corresponded to a porosity of approximately 97% (Table 2). Comparable data have been reported previously for cryogels obtained after the dissolution of cellulose II in calcium thiocyanate (0.060 g/cm^3^) [47], for cellulose aerogels made from microfibrillated cellulose (0.032–0.079 g/cm^3^) [48], and for cellulose cryogels made from microcrystalline cellulose (0.070 g/cm^3^) [49]. For a sample with the highest density of 0.144 g/cm^3^, the porosity was the lowest (89.6%).

The stability of cryogels in water was evaluated (SR, Table 2). Samples 1 and 2 retained their shape after being kept in water for 24 h. The highest SR value of 8.7 g/g was characteristic of the sample obtained by dissolving cellulose at 20 °C for 24 h. This value decreased by half (4.4 g/g, sample 2) with an increased dissolution time of cellulose under similar conditions (cellulose concentration of 5%, dissolution temperature of 20 °C). Comparable values were obtained previously (5.5 and 3.5 g/g) for cryogels based on MCC/PVA (50/50) [30], and values of 5.8 and 4.7 g/g were obtained for cryogels based on NaCMC and PVA, respectively [32]. Sample 4 lost its shape immediately upon immersion in water. Thus, minimal destruction under mild conditions of dissolution at a temperature of 20 °C allows for obtaining the most stable cryogels.

The dissolution of cellulose at a temperature of 20 °C and a dissolution time of 24 h allowed for obtaining a cryogel with a minimal destruction of cellulose and the highest specific surface area, at 38.1 m^2^/g (sample 1, Table 2). An increase in the dissolution time to 46 h, corresponding to a 1.8-fold decrease in M_w_, led to a more than 8-fold decrease in the specific surface area. The sample obtained by dissolving cellulose upon heating had a specific surface area that was six times lower. Thus, the dissolution of cellulose at a temperature of 20 °C and a dissolution time of 24 h constituted the best conditions for obtaining a cryogel with a high specific surface area.

### 3.2. Crystallinity of Cellulose and Cellulose Cryogels

**Figure 1:**
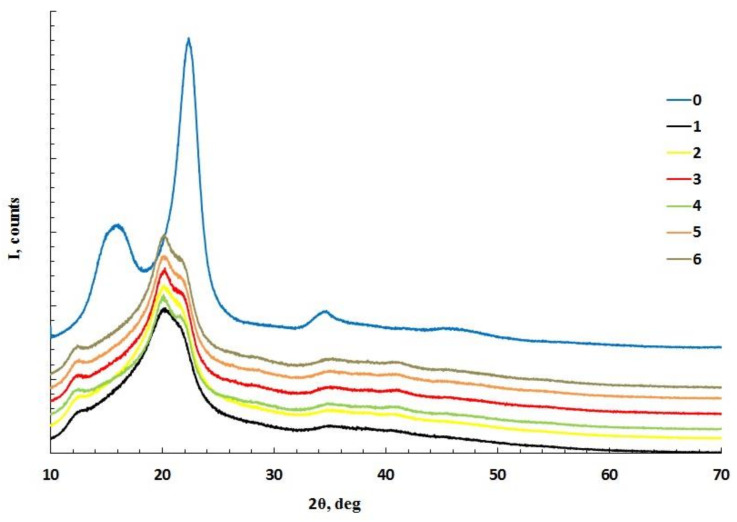
Diffractograms of microcrystalline cellulose (0) and cellulose cryogel samples (numbers according to Table 1).

Diffractograms of MCC and cellulose cryogels are shown in Figure 1. The diffraction pattern for MCC corresponds to the diffractogram for cellulose I, while the diffraction patterns for cellulose cryogels correspond to the diffractograms for cellulose II [50]. In one of the studies, it was shown that at dissolution temperatures of 30 and 50 °C, the crystallinity degree of cellulose decreased with increasing dissolution time. At higher temperatures (70 °C) and a longer amount of time, the decrystallization effect decreased [51]. In our case, the crystallinity of cellulose after dissolution in phosphoric acid and regeneration with water decreased by about twofold (Table 1). The maximum crystallinity was obtained for cellulose dissolution at room temperature.

### 3.3. FTIR Spectroscopy

**Figure 2:**
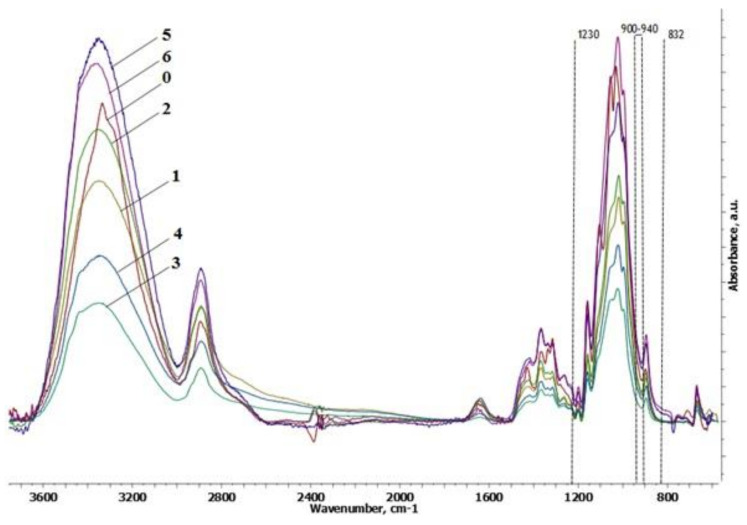
FTIR spectra of microcrystalline cellulose (0) and cellulose cryogel samples (numbers according to Table 1).

Similar to a previous study [19], the rearrangement of hydrogen bonds in cellulose was determined in the cryogel spectra (Figure 2). This involved changes in primary (1075–1000 cm^−1^) and secondary (1125–1030 cm^−1^) hydroxyl regions and the absorption band at 3000–3600 cm^−1^ compared to initial MCC. An increase in the number of reducing groups and aldehyde groups was also observed (increased intensity at 1650 and 2700 cm^−1^). Bands characteristic of the phosphorylation reaction (1230 cm^−1^ (P=O), 900–940 cm^−1^ (P–OH), and 832 cm^−1^ (aliphatic P–O–C)) [52] in the spectra of the cryogels were absent. Thus, the phosphorylation of the cellulose after dissolution in phosphoric acid was not detected.

### 3.4. Mechanical Properties

Mechanical tests were carried out for samples that retained their shape (samples 1, 2, and 4). Initial tests showed that the samples were not destroyed at high compression ratios, except for sample 4. Therefore, the compression strain limit was set to 70%. During the tests, the following characteristics of the cryogels were determined: compressive modulus (E), yield stress (σ_y_), and stress corresponding to the critical deformation of 70% (σ_70_). The test results are presented in Table 3.

For a cryogel sample obtained by dissolving cellulose at 20 °C for a dissolution time of 24 h (sample 1), the highest mechanical strength was obtained (Table 3). Previously, for cryogels based on MCC (from 30 to 80%) with the addition of PVA, a similar E modulus was obtained (from 800 to 1200 Pa) [30]. A dissolution time that was two times higher, along with a significant degradation of cellulose (i.e., 3.4-fold decrease in the molecular weight), led to an almost 10-fold decrease in the compressive modulus (sample 2). The compressive modulus of the resulting cryogels, however, was higher than for glass-reinforced nanocellulose cryogels (24 kPa) [35]. Heating during the dissolution led to a 4.2-fold decrease in the molecular weight of cellulose, and resulted in the instant destruction of the sample during the mechanical test (sample 4). Thus, to obtain the cryogel with the highest-strength cellulose dissolution, a temperature of 20 °C should be applied.

### 3.5. Morphology of Cellulose Cryogels

**Figure 3:**
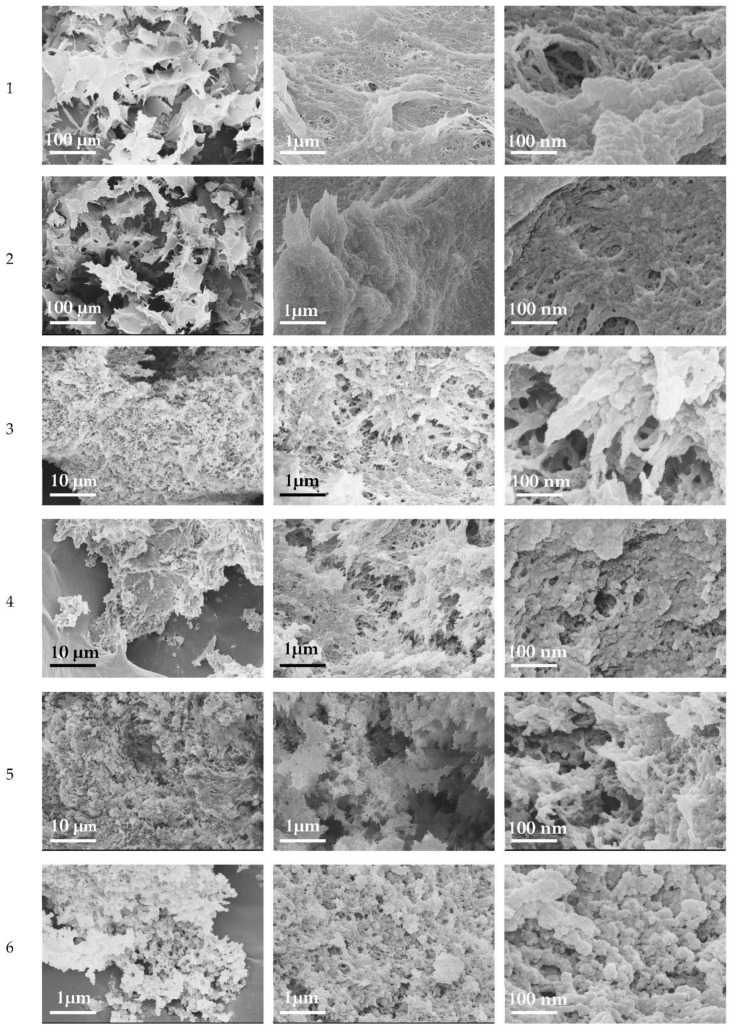
SEM images of cryogel samples 1–6.

Figure 3 shows the morphology of the obtained cryogel samples. After dissolution in acid, cellulose is able to rebuild crystalline structures in the form of nanoparticles and plates. The formation of spherical crystallites of cellulose with a size of 10–100 nm has been previously observed [53,54].

Scanning electron microscopy images for all obtained cryogel samples revealed netted structures, plates up to 1 μm in size, and spherical structures of cellulose with a size of 20–100 nm. The pore diameter was less than 1 μm. It has been noted [55] that the porous surface of PVA/cellulose-based cryogels with pore sizes up to 7 µm is beneficial for wound dressings, as increased oxygen levels in the tissue stimulate the epithelialization and fibroblast proliferation. For the nanocellulose cryogel the pore size was 140 μm; however, this proved to be suitable for the adhesion and proliferation of osteoblasts without cell aggregation and the proper vascularization of the scaffold [35]. Cryogels obtained after dissolving cellulose with heating had a less dense structure (samples 3–6). The bigger plate structures that were tens of μm in size consisted of smaller plate structures of up to 1 μm, which had numerous pores of submicron and a nanoscale diameters of up to 1 μm. Smaller plate structures consisted of spherical particles with a diameter of 20–100 nm. Similar spherical crystals were previously reported for cellulose after exhaustive acidic hydrolysis. Cellulose with lower M_w_ formed more pronounced spherical particles (Figure 3, samples 3, 5, and 6) and had a less dense structure.

### 3.6. Effects of Freezing Time on the Properties of Cryogels

Freezing parameters are known to affect the properties of the cryogels [11,14]. We increased the freezing time of cellulose hydrogels from 3 days (samples 1–6) to 10 (sample 7) and 28 days (sample 8). These samples were prepared after the dissolution of MCC at 20 °C during 24 h, similar to sample 1, which had the best mechanical properties, stability in water, and maximum specific surface area. The properties of the obtained cryogels are presented in Table 4.

**Figure 4:**
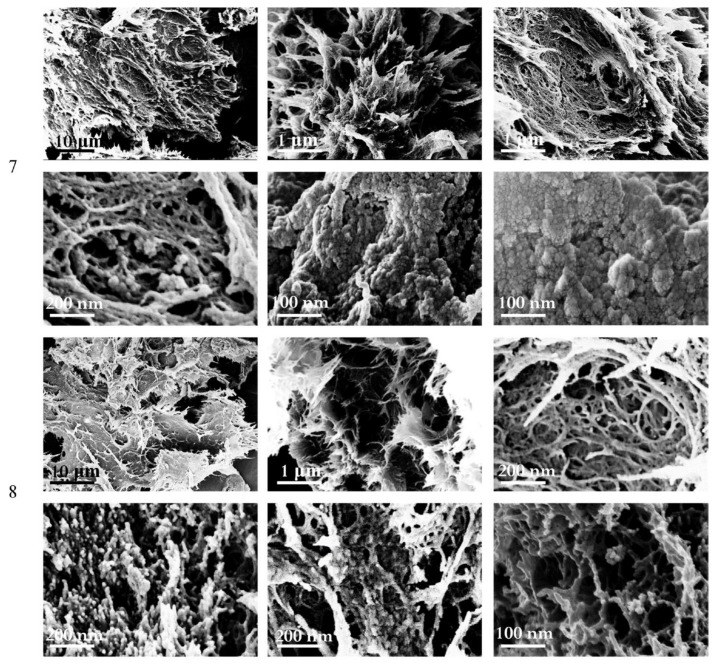
SEM images of cryogel samples 7 and 8.

Long freezing leads to the formation of denser cryogel structures. Shrinkage was significantly increased, as was the density, resulting in reduced porosity and swelling properties compared to previous samples. The mechanical properties after 28 days of freezing were improved (Young’s modulus E increased by more than twofold for sample 8). Therefore, the rearrangement of cellulose macromolecules in gel structures can occur. Prolonged freezing led to a decrease in the specific surface area of samples 7 and 8. At the same time, a larger value was noted for sample 8, which was possibly due to the formation of a pronounced network structure (confirmed by the SEM images, Figure 4) compared to sample 7. At the same time, the obtained cryogels were characterized by the presence of lamellar structures and clusters of spherical structures (Figure 4).

### 3.7. Composite Cryogels

Previously, it was shown that the inclusion of chitin or cellulose in the matrix in the form of nanowhiskers leads to changes in the strength of the producing materials [56,57,58,59]. CNWs with hydrodynamic radii of 15 ± 2 nm and 114 ± 28 nm and a ζ-potential of +(23.3 ± 0.5) mV were used as nanofillers for cellulose cryogels. Positively charged amino groups of deacetylated chitin can form a polyelectrolyte complex with negatively charged groups of the matrix, acting as a natural crosslinking agent. The incorporation of such nanowhiskers into the polysaccharide matrix enhances the strength, rigidity, and stability of the hydrogel structure, which at the micro-level leads to increased cell growth on composite scaffolds [60]. Composite cryogels were obtained by dissolving cellulose in phosphoric acid at a temperature of 20 °C for 24 h; the cellulose concentration in the solution was 5%, and CNWs were introduced into the gel at the stage of cellulose regeneration from a phosphoric acid solution. The characteristics of the composite cryogels were determined according to the methods presented in [19]. The results are presented in Table 5.

The yield of composite cryogels increased with an increase in the amount of introduced CNWs (samples 9–11 and 14). Lower yields of cryogels (samples 12 and 13) were associated with greater losses at the stage of washing regenerated cellulose from phosphoric acid. The density of composite cryogels (samples 9–14) was higher than the density of a cryogel without CNWs (sample 1). The incorporation of the CNWs contributed to a denser shrinkage of the samples; the volumes of composite cryogels were smaller than the volumes of cryogels without CNWs. Thus, the incorporation of CNWs led to the formation of a denser cryogel structure.

All samples had positive volume changes (∆V), indicating the swelling of the cellulose after coagulation. In this case, the samples with the largest amounts of incorporated CNWs (samples 11 and 12) had the highest positive values. The incorporation of CNWs at amounts of 5 and 10% led to a greater absorption of water, along with the swelling of the corresponding cellulose hydrogels. With an increase in density, the values of the swelling ratio for composite cryogels decreased. The denser structure of cryogels prevented the penetration of water molecules into the polymer network. The specific surface area for composite cryogels (samples 9–14) was lower than for unfilled cellulose cryogels.

The mechanical properties of composite cryogels (compressive modulus, E) improved with the addition of 1% and 2.5% CNWs (samples 9 and 10, respectively). The addition of 5% and 10% CNWs (samples 11 and 12, respectively) led to a decrease in the compressive modulus. This was possibly due to the aggregation of CNWs, which prevented their uniform distribution in the cryogel and redistribution of stress under mechanical loads, and also reduced the mobility of the cellulose molecules. An increase in freezing time up to 10 days led to a threefold increase in the compressive modulus. Thus, there is an optimal amount of nanofiller to be introduced to improve the mechanical properties of the cryogel. For higher-strength cryogels, the freezing time of cellulose hydrogels can be increased.

**Figure 5:**
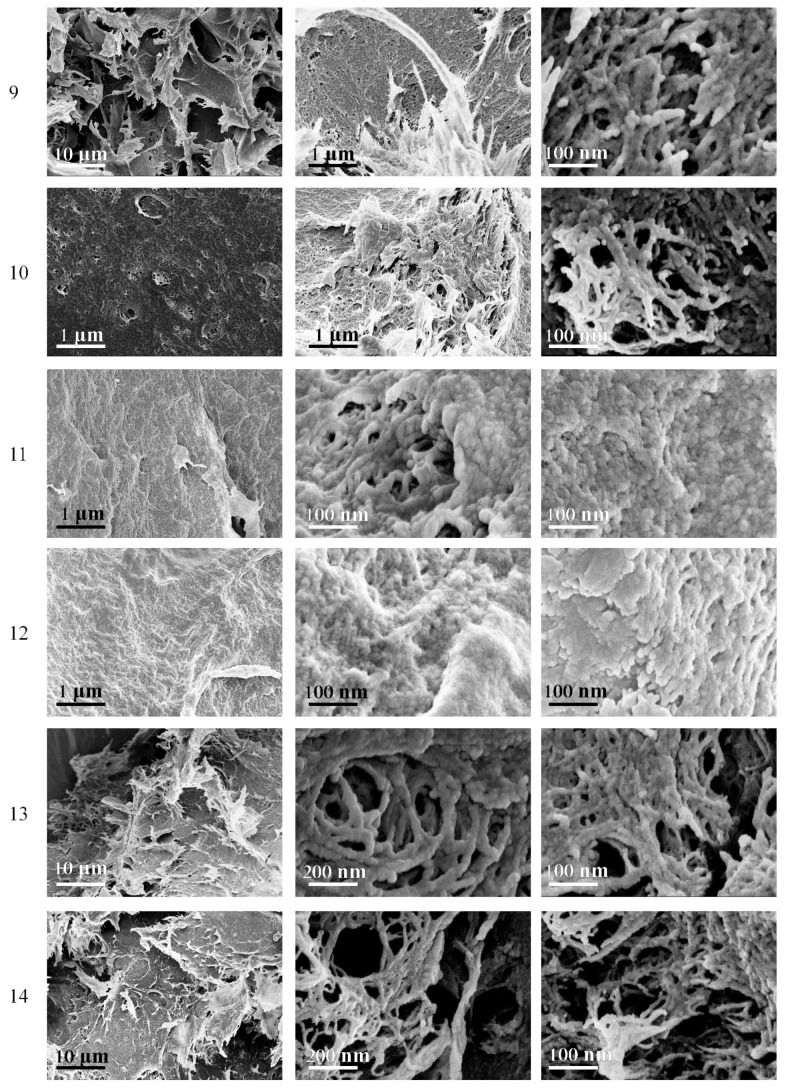
SEM images of cryogel samples 9–14.

The morphology of composite cryogels (Figure 5) is similar to the morphology of cellulose cryogels presented above (Figure 3 and Figure 4). Long freezing led to the formation of a predominant network structure (samples 13 and 14).

### 3.8. Biocompatibility of Cryogels

Sample 1 was unstable in the medium used to evaluate biocompatibility. Conversely, samples 7, 8, and 13 were stable. For the latter, biocompatibility studies were continued. A significant swelling of the materials was noted after 3 days of the co-culture of samples with cells. Meanwhile, samples 7 and 8 were fragmented during the manipulations. Sample 13 was denser, and no fragmentation was observed during the manipulations.

The cells on the coverslips of the control group were spread out on the surface of the glass with the formation of a subconfluent monolayer, and had a typical elongated shape with multiple processes. Some of the cells seemed to be proliferating. Longitudinal linear structures that are stained red (i.e., actin filaments) were clearly visible in the cells.

**Figure 6:**
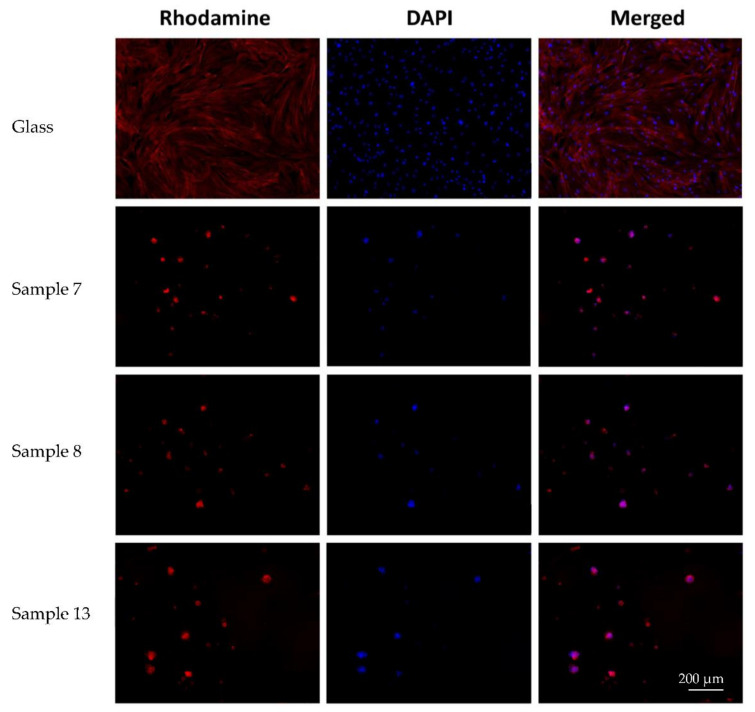
Morphology of mesenchymal stem cells adhered on coverslips and on experimental samples (magnification of ×10).

The microscopic picture of cells on the surface of all studied samples differed insignificantly. On all experimental samples, MSCs were located superficially, and formed spheroid colonies (mainly) or separately adhered single cells. MSCs were rounded/nearly rounded, with the diffuse staining of the cytoskeleton with phalloidin (Figure 6). The cell area was significantly smaller compared to MSCs on the surface of the coverslips. There were no obvious signs of cell migration or proliferation along the periphery of the spheroid colonies.

**Figure 7:**
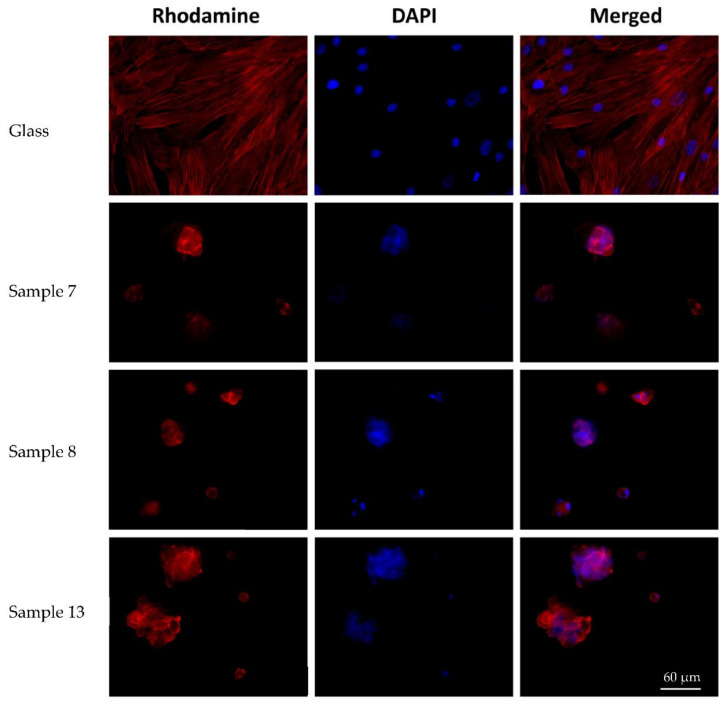
Spheroids on the surface of the cryogel samples (magnification of ×40).

In the course of the quantitative analysis, the number of spheroids was counted. Their maximum longitudinal dimensions and the maximum depth of colonies on the surface of the samples were measured, taking into account the surface morphology. Cells were not counted, due to the impossibility of accurately determining the numbers of nuclei in spheroid colonies. The number of spheroids on the samples’ surfaces and the maximum depth of their location did not differ significantly for samples 7 and 8 (*p* > 0.05) (Table 6, Figure 7). Meanwhile, the sizes of spheroids on sample 13 were significantly (*p* < 0.05) larger than on the other two types of materials (*p* < 0.05).

Despite MSCs being 2D adherent cultures, the spheroid formation on the materials’ surfaces seemed to be significant. We demonstrated that the adherent abilities of MSCs were not observed on cryogels, whereas the formation of spheroidal cell aggregates or spheroids was observed mainly on the samples with CNWs. The addition of CNWs into the cellulose cryogels led to a change in the properties of the material. The cryogel became denser and more suitable for manipulation even after 3 days of co-culture with cells. In addition, the inclusion of CNWs in the cryogel structure led to an improvement in biocompatibility. This was evidenced by an increase in the size of cellular spheroids on the sample surface.

It has been demonstrated that material properties affect the 2D (monolayer) or 3D (spheroids) type of MSC cultures. In particular, Young’s elasticity modulus of the materials surrounding the cells is an important characteristic. The cells in 2D monolayers reside on the material with an elasticity modulus in the GPa range, whereas spheroids should be surrounded by cells and materials with a combined elasticity modulus of less than 0.1 kPa [61,62].

The process of spheroid formation and the ability of the material to form them on their surface differ widely, and depend on the physical and chemical properties of the materials. In particular, it is more pronounced on chitosan membranes [63,64]. It appears that adding CNWs to the cryogels also led to an increased ability to form MSC spheroids on the sample surface, but not on the 2D monolayer.

MSC spheroids can provide multiple effects. They enhance paracrine secretion of angiogenic, anti-tumorigenic, and pro- and anti-inflammatory factors. Spheroids improve cell survival, increase differentiation potential, and delay replicative senescence of MSCs [62,65,66]. Thus, 3D MSC spheroids can be a useful tool in therapy because of their enhanced angiogenic, anti-tumorigenic, or tissue repair and regenerative effects [66,67,68]. Despite the low cell-adherent ability of cryogels, their cell spheroid formation properties will probably be demanded in clinical applications.

## 4. Conclusions

Ultralight (0.046–0.162 g/cm^3^) and highly porous (88.2–96.7%) cellulose-based cryogels were obtained using phosphoric acid as a solvent, and were characterized. It was established that the contact of cellulose with acid should be minimized and carried out under mild conditions. The cryogel with the best characteristics was obtained from a solution with a cellulose concentration of 5%, a dissolution temperature of 20 °C, and a dissolution time of 24 h. These conditions allowed for less decrease in molecular weight. The M_w_ of cellulose in all samples decreased by 2–8-fold, affecting gel shrinkage and pore formation. Increasing the temperature or exposure time to acid resulted in higher cellulose destruction and the production of cryogel structures with poor surface area, porosity, swelling capacity, mechanical properties, and stability. Regeneration resulted in cryogels with varied morphology. Spherical structures of cellulose with a size of 20–100 nm, netted structures, and plates up to 1 μm in size were observed. XRD showed that the regenerated cellulose in the cryogels had a cellulose II structure with crystallinity of 26.8–28.4% (two times lower compared to the initial cellulose). FTIR spectroscopy also revealed a decrease in the number of hydrogen bonds after the regeneration of cellulose from the phosphoric acid solutions. It was shown that prolonged (2–4 weeks) freezing resulted in denser and stronger cryogels with a predominant network structure, and these cryogels were stable enough to evaluate the biocompatibility. The incorporation of CNWs into cellulose cryogels led to a threefold increase in strength and better cytocompatibility.

## Figures and Tables

**Table 1:** Dissolution conditions and characteristics of cellulose cryogels.

Sample	Cellulose Concentration, %	Dissolution Time, h	Temperature, °C	Sample Shape	Yield, %	∆ V, %	$Q_{max},$ %	Mw (Ð *)	Crystallinity, %
1	5	24	20 ± 2	Intact cylindrical	93.6	60	3319	53,770 (3.8)	28.2
2	5	46			86.2	60	3647	29,620 (2.6)	28.4
3	5	24	29 ± 1	Partially cylindrical and powder	88.4	−45	1189	18,110 (2.7)	28.0
4	10	24		Intact cylindrical	90.9	17.5	1210	24,000 (3.0)	27.0
5	5	24	39 ± 1	Partially cylindrical and powder	90.3	−34	1388	19,390 (2.3)	27.7
6	5	48			86.1	−62.5	803	13,190 (2.4)	27.9

* Ð = M_w_/M_n_ is the dispersity, where M_w_ is the weight-average molecular weight and M_n_ is the number-average molecular weight.

**Table 2:** Characterization of intact cylindrical cryogels.

Sample	Cryogel Volume, cm^3^	Volume Shrinkage, %	ρ, g/cm^3^	Porosity, %	SR, g/g	Specific Surface Area, m^2^/g
1	4.5	10.7	0.052	96.2	8.7	38.1
2	4.7	6.9	0.046	96.7	4.4	4.6
4	12.6	37.3	0.144	89.6	Sample broken	6.0

**Table 3:** Mechanical properties of cellulose cryogels.

Sample	E, kPa	σ_y_, kPa	σ_max_, kPa	ε_max_, %
1	1231 ± 132	79 ± 26	366 ± 45	70
2	126 ± 25	43 ± 11	268 ± 30	70
4	Broken under load			

**Table 4:** Effects of freezing time on the properties of cryogels.

Sample	Freezing Time, Days	Yield, %	Cryogel Volume, cm^3^	Volume Shrinkage, %	ρ, g/cm^3^	Porosity, %	∆ V, %	$Q_{max},$ %	SR, g/g	E, kPa	Crystallinity, %	Specific Surface Area, m^2^/g
1	3	93.6	4.5	10.7	0.052	96.2	60	3319	8.7	1231 ± 132	28.2	38.062
7	10	90.0	1.43	71.4	0.157	88.6	66	3566	7.1	1099 ± 121	27.4	4.647
8	28	91.2	1.40	71.9	0.162	88.2	60	3336	5.1	2872 ± 478	26.8	16.765

**Table 5:** Conditions of the production process and the characteristics of composite cryogels.

Sample	CNW, %	Freezing Time, Days	Yield, %	Cryogel Volume, cm^3^	Volume Shrinkage, %	∆ V, %	ρ, g/cm^3^	Porosity, %	$Q_{max},$ %	SR, g/g	Specific Surface Area, m^2^/g	E, kPa
1	0	3	93.6	4.50	10.7	60	0.052	96.2	3319	8.7	38.062	1231 ± 132
9	1	3	89.2	1.84	63.2	40	0.121	91.2	3010	4.9	0.981	1238
10	2.5	3	90.8	2.76	44.9	40	0.082	94.0	2946	7.7	2.629	1570
11	5	3	92.6	3.10	37.8	124	0.078	94.3	4529	6.6	1.012	273
12	10	3	83.3	2.49	50.2	104	0.092	93.3	4349	6.8	0.530	383 ± 150
13	1	10	75.6	1.80	63.8	60	0.106	92.3	4144	4.6	9.737	3120 ± 120
14	2.5	10	90.5	2.51	49.8	64	0.092	93.3	3567	4.9	0.935	3050 ± 160

**Table 6:** Characteristics of spheroids on the tested cryogel samples (mean ± SE).

Sample	Number of Spheroids/mm^2^	Spheroid Size, µm	Depth of Spheroids Location, µm
7	7.3 ± 0.9	41.0 ± 1.4	223 ± 5
8	6.6 ± 1.8	44 ± 2	230 ± 5
13	8.6 ± 0.7	57 ± 3 *	230 ± 9

* *p* < 0.05 comparing sample 7 and sample 8.

## Data Availability

Data is contained within the article.
